# (*R*)-[1-(2-Chloro­phen­yl)-2-meth­oxy-2-oxoeth­yl][2-(thio­phen-2-yl)eth­yl]ammonium (+)-camphor-10-sulfonate acetone monosolvate

**DOI:** 10.1107/S160053681004523X

**Published:** 2010-11-10

**Authors:** Yan-Shu Liang, Shuai Mu, Ying Liu, Deng-Ke Liu

**Affiliations:** a Tianjin University of Commerce, Tianjin 300134, People’s Republic of China; b School of Chemical Engineering and Technology, Tianjin University, Tianjin 300072, People’s Republic of China; c Tianjin Institute of Pharmaceutical Research, Tianjin 300193, People’s Republic of China

## Abstract

The title compound, C_15_H_17_ClNO_2_S^+^·C_10_H_15_O_4_S^−^·C_3_H_6_O, was synthesized by *N*-alkyl­ation of α-amino-(2-chloro­phen­yl)acetate with 2-thienylethyl *p*-toluene­sulfonate, followed by reaction with (+)-camphor-10-sulfonic acid. In the crystal, the cations and anions are linked through N—H⋯O hydrogen bonds. The thio­phene ring of the cation was found to be disordered over two sites, with refined occupancies of 0.798 (4) and 0.202 (4).

## Related literature

For background to the anti­platelet agent clopidogrel, see: Kang *et al.* (2007 ▶). For the preparation of the title compound, an inter­mediate of clopidogrel, see: Descamps & Radisson (1992 ▶). For a database of bond lengths and angles, see: Bruno *et al.* (2004 ▶).
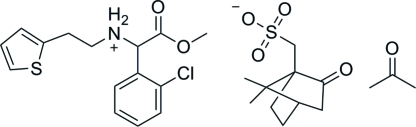


## Experimental

### 

#### Crystal data

C_15_H_17_ClNO_2_S^+^·C_10_H_15_O_4_S^−^·C_3_H_6_O
*M* *_r_* = 600.16
Orthorhombic, 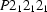

*a* = 12.101 (3) Å
*b* = 14.209 (4) Å
*c* = 18.325 (5) Å
*V* = 3150.7 (14) Å^3^
*Z* = 4
Mo *K*α radiation
μ = 0.30 mm^−1^
*T* = 294 K
0.26 × 0.24 × 0.20 mm

#### Data collection

Bruker SMART-CCD area-detector diffractometer
Absorption correction: multi-scan (*SADABS*; Sheldrick, 1996 ▶) *T*_min_ = 0.912, *T*_max_ = 0.943
18154 measured reflections
6435 independent reflections
4010 reflections with *I* > 2σ(*I*)
*R*_int_ = 0.039

#### Refinement

*R*[*F* ^2^ > 2σ(*F* ^2^)] = 0.039
*wR*(*F* ^2^) = 0.096
*S* = 0.99
6435 reflections
402 parameters
70 restraints
H atoms treated by a mixture of independent and constrained refinement
Δρ_max_ = 0.17 e Å^−3^
Δρ_min_ = −0.18 e Å^−3^
Absolute structure: Flack (1983 ▶), 2828 Friedel pairs
Flack parameter: −0.04 (6)

### 

Data collection: *SMART* (Bruker, 1997 ▶); cell refinement: *SAINT* (Bruker, 1997 ▶); data reduction: *SAINT*; program(s) used to solve structure: *SHELXS97* (Sheldrick, 2008 ▶); program(s) used to refine structure: *SHELXL97* (Sheldrick, 2008 ▶); molecular graphics: *SHELXTL* (Sheldrick, 2008 ▶); software used to prepare material for publication: *SHELXTL*.

## Supplementary Material

Crystal structure: contains datablocks global, I. DOI: 10.1107/S160053681004523X/bh2318sup1.cif

Structure factors: contains datablocks I. DOI: 10.1107/S160053681004523X/bh2318Isup2.hkl

Additional supplementary materials:  crystallographic information; 3D view; checkCIF report

## Figures and Tables

**Table 1:** Hydrogen-bond geometry (Å, °)

*D*—H⋯*A*	*D*—H	H⋯*A*	*D*⋯*A*	*D*—H⋯*A*
N1—H1*B*⋯O3	0.93 (3)	1.82 (3)	2.729 (3)	164 (3)
N1—H1*A*⋯O5^i^	0.88 (3)	2.63 (2)	3.180 (3)	121.1 (19)
N1—H1*A*⋯O4^i^	0.88 (3)	1.99 (3)	2.856 (3)	169 (2)

Symmetry code: (i) 
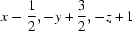
.

**Fig. 1.**
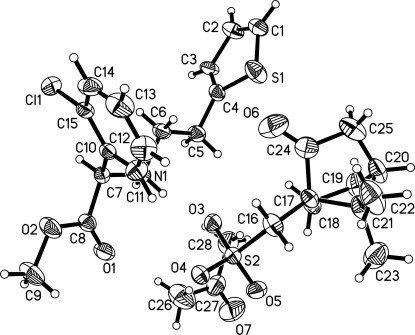
The molecular structure of (I), with the atom-numbering scheme and 30% probability displacement ellipsoids.

**Fig. 2.**
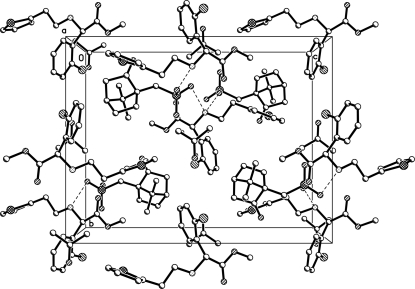
Packing diagram for (I) with hydrogen bonds drawn as dashed lines.

## supplementary crystallographic information

### Comment

Clopidogrel treatment is associated with a reduction in thrombotic complications in coronary stent placement, improved outcome after acute coronary syndromes, and decreased mortality in patients with coronary artery disease (Kang *et al.*, 2007). Now, we present the crystal structure of the title compound, (I), an intermediate of Clopidogrel (Fig. 1, Table 1).

All bond lengths and angles in (I) are within normal ranges (Bruno *et al.*, 2004). In the cation, C5 to C9, N1, O1 and O2 are almost coplanar (r.m.s. deviation 0.0363 Å and maximum deviation 0.0610 (2) Å). In the anion, the dihedral angles formed between C17 to C20 plane and C17/C20/C21 plane, C17/C20/C21 plane and C17/C20/C24/C25 plane are 54.45 (2)° and 54.47 (3)°, respectively. The crystal is stabilized by N—H···O hydrogen bonds between the cations and the anions, which link the molecules into sheets in the *bc* plane (Fig. 2, Table 2).

### Experimental

(I) was prepared according to the method of Descamps & Radisson (1992). 12.5 g of methyl alpha-amino(2-chlorophenyl)acetate was released from its hydrochloride by reaction with 9 g of NaHCO_3_ in the presence of 200 ml of CH_2_Cl_2_ and 65 ml of water. The resulting amine was dissolved in 65 ml of acetonitrile; 6.2 g of NaHCO_3_ and 15.6 g of 2-thienylethyl-*para*-toluenesulfonate were then introduced; the mixture was kept at 353 K for 22 h and the volatile products were then evaporated off under reduced pressure. The residue was dissolved in 200 ml of ethyl acetate and 65 ml of water; the organic phase was separated off. After the usual treatments the aminoester was obtained and dissolved in 70 ml of acetone, then 8.7 g of (+)-10-camphorsulfonic acid was introduced. The mixture was stirred at room temperature for 12 h to get precipitate, and 11 g of camphorsulfonate was thus obtained. The camphorsulfonate was suspended in 50 ml of refluxing acetone and 25 ml of methyl ethyl ketone to obtain complete dissolution. The mixture was standing under 298 K, then white crystals were grown slowly. The crystals were washed with cold acetone, yield 6.8 g, m.p. 368 K.

### Refinement

2828 Friedel pairs were used in the Flack parameter refinement. The thiophene ring was found to be disordered and was refined with the restraints d(C—S) = 1.72 Å, d(C—C) = 1.50 Å and d(C═C) = 1.34 Å, respectively. Ammonium H atoms were initially located in a difference map and refined with the restraints N—H = 0.85 (2) Å. Other H atoms were positioned geometrically and refined using a riding model, with d(C—H) = 0.93–0.98 Å and *U*_iso_(H) = 1.2*U*_eq_(C) or 1.5*U*_eq_(C_methyl_).

### Crystal data

**Table:**

C_15_H_17_ClNO_2_S^+^·C_10_H_15_O_4_S^−^·C_3_H_6_O	*D*_x_ = 1.265 Mg m^−^^3^
*M**_r_* = 600.16	Melting point: 368 K
Orthorhombic, *P*2_1_2_1_2_1_	Mo *K*α radiation, λ = 0.71073 Å
Hall symbol: P 2ac 2ab	Cell parameters from 4370 reflections
*a* = 12.101 (3) Å	θ = 2.2–23.4°
*b* = 14.209 (4) Å	µ = 0.30 mm^−^^1^
*c* = 18.325 (5) Å	*T* = 294 K
*V* = 3150.7 (14) Å^3^	Block, colourless
*Z* = 4	0.26 × 0.24 × 0.20 mm
*F*(000) = 1272	

### Data collection

**Table:**

Bruker SMART-CCD area-detector diffractometer	6435 independent reflections
Radiation source: fine-focus sealed tube	4010 reflections with *I* > 2σ(*I*)
graphite	*R*_int_ = 0.039
φ and ω scans	θ_max_ = 26.5°, θ_min_ = 1.8°
Absorption correction: multi-scan (*SADABS*; Sheldrick, 1996)	*h* = −14→15
*T*_min_ = 0.912, *T*_max_ = 0.943	*k* = −17→17
18154 measured reflections	*l* = −13→22

### Refinement

**Table:**

Refinement on *F*^2^	Secondary atom site location: difference Fourier map
Least-squares matrix: full	Hydrogen site location: inferred from neighbouring sites
*R*[*F*^2^ > 2σ(*F*^2^)] = 0.039	H atoms treated by a mixture of independent and constrained refinement
*wR*(*F*^2^) = 0.096	*w* = 1/[σ^2^(*F*_o_^2^) + (0.0457*P*)^2^] where *P* = (*F*_o_^2^ + 2*F*_c_^2^)/3
*S* = 0.99	(Δ/σ)_max_ = 0.003
6435 reflections	Δρ_max_ = 0.17 e Å^−^^3^
402 parameters	Δρ_min_ = −0.18 e Å^−^^3^
70 restraints	Absolute structure: Flack (1983), 2828 Friedel pairs
0 constraints	Flack parameter: −0.04 (6)
Primary atom site location: structure-invariant direct methods	

### Fractional atomic coordinates and isotropic or equivalent isotropic displacement parameters (Å^2^)

**Table:**

	*x*	*y*	*z*	*U*_iso_*/*U*_eq_	Occ. (<1)
Cl1	0.41386 (6)	0.37358 (5)	0.48307 (4)	0.0722 (2)	
S2	0.77423 (6)	0.77371 (5)	0.58832 (4)	0.04884 (18)	
O1	0.60127 (19)	0.69806 (14)	0.40519 (11)	0.0766 (6)	
O2	0.62818 (19)	0.55299 (14)	0.36419 (11)	0.0779 (7)	
O3	0.65698 (14)	0.75282 (13)	0.58489 (10)	0.0617 (5)	
O4	0.83735 (15)	0.72491 (13)	0.53212 (9)	0.0610 (5)	
O5	0.79800 (15)	0.87314 (12)	0.58933 (11)	0.0653 (5)	
O6	0.6787 (2)	0.60281 (18)	0.75506 (17)	0.1049 (9)	
N1	0.4941 (2)	0.64436 (14)	0.52533 (12)	0.0421 (5)	
S1	0.40689 (11)	0.62354 (14)	0.78089 (8)	0.0841 (6)	0.798 (4)
C1	0.2895 (5)	0.5925 (5)	0.8266 (3)	0.0692 (18)	0.798 (4)
H1	0.2888	0.5652	0.8727	0.083*	0.798 (4)
C2	0.1991 (5)	0.6124 (5)	0.7880 (3)	0.0622 (16)	0.798 (4)
H2	0.1275	0.6025	0.8047	0.075*	0.798 (4)
C3	0.2257 (5)	0.6518 (10)	0.7158 (5)	0.0578 (19)	0.798 (4)
H3	0.1731	0.6706	0.6819	0.069*	0.798 (4)
C4	0.3347 (2)	0.65728 (18)	0.70495 (14)	0.0510 (7)	0.798 (4)
S1'	0.1970 (7)	0.6383 (13)	0.7155 (7)	0.082 (3)	0.202 (4)
C1'	0.2174 (16)	0.594 (2)	0.8011 (11)	0.058 (8)	0.202 (4)
H1'	0.1667	0.5567	0.8258	0.069*	0.202 (4)
C2'	0.3154 (17)	0.6201 (16)	0.8281 (7)	0.050 (6)	0.202 (4)
H2'	0.3368	0.6123	0.8765	0.060*	0.202 (4)
C3'	0.3863 (12)	0.6634 (16)	0.7701 (6)	0.077 (8)	0.202 (4)
H3'	0.4553	0.6904	0.7777	0.092*	0.202 (4)
C4'	0.3347 (2)	0.65728 (18)	0.70495 (14)	0.0510 (7)	0.202 (4)
C5	0.3906 (2)	0.69093 (18)	0.63647 (15)	0.0569 (7)	
H5A	0.4520	0.7316	0.6494	0.068*	
H5B	0.3387	0.7276	0.6079	0.068*	
C6	0.4329 (2)	0.60951 (17)	0.59081 (14)	0.0534 (7)	
H6A	0.4816	0.5707	0.6202	0.064*	
H6B	0.3712	0.5709	0.5752	0.064*	
C7	0.5266 (2)	0.56885 (16)	0.47250 (14)	0.0444 (6)	
H7	0.4588	0.5425	0.4517	0.053*	
C8	0.5905 (2)	0.61535 (19)	0.41131 (14)	0.0496 (6)	
C9	0.6918 (3)	0.5885 (2)	0.30261 (18)	0.0890 (12)	
H9A	0.6464	0.6296	0.2738	0.133*	
H9B	0.7162	0.5367	0.2731	0.133*	
H9C	0.7548	0.6226	0.3203	0.133*	
C10	0.5895 (2)	0.48914 (16)	0.50914 (14)	0.0430 (6)	
C11	0.6942 (2)	0.50530 (19)	0.53687 (16)	0.0614 (8)	
H11	0.7251	0.5650	0.5332	0.074*	
C12	0.7536 (3)	0.4337 (2)	0.56997 (18)	0.0763 (10)	
H12	0.8237	0.4456	0.5886	0.092*	
C13	0.7088 (3)	0.3454 (2)	0.5751 (2)	0.0829 (10)	
H13	0.7489	0.2975	0.5974	0.100*	
C14	0.6057 (3)	0.3269 (2)	0.54794 (18)	0.0684 (9)	
H14	0.5760	0.2666	0.5513	0.082*	
C15	0.5459 (2)	0.39861 (18)	0.51539 (14)	0.0506 (7)	
C16	0.8220 (2)	0.7252 (2)	0.67130 (14)	0.0610 (7)	
H16A	0.8147	0.6573	0.6679	0.073*	
H16B	0.9004	0.7388	0.6750	0.073*	
C17	0.7675 (2)	0.75628 (18)	0.74299 (13)	0.0502 (7)	
C18	0.6771 (3)	0.8337 (2)	0.74243 (16)	0.0683 (9)	
H18A	0.6980	0.8854	0.7107	0.082*	
H18B	0.6068	0.8084	0.7262	0.082*	
C19	0.6703 (3)	0.8666 (3)	0.82296 (18)	0.0803 (10)	
H19A	0.6923	0.9319	0.8279	0.096*	
H19B	0.5963	0.8587	0.8423	0.096*	
C20	0.7528 (3)	0.8007 (3)	0.86121 (17)	0.0772 (10)	
H20	0.7777	0.8227	0.9092	0.093*	
C21	0.8457 (2)	0.7907 (2)	0.80388 (15)	0.0661 (8)	
C22	0.9343 (3)	0.7182 (3)	0.8242 (2)	0.1106 (14)	
H22A	0.9014	0.6568	0.8268	0.166*	
H22B	0.9657	0.7341	0.8707	0.166*	
H22C	0.9913	0.7182	0.7877	0.166*	
C23	0.9031 (3)	0.8840 (3)	0.7847 (2)	0.1080 (13)	
H23A	0.9479	0.9039	0.8250	0.162*	
H23B	0.8483	0.9311	0.7746	0.162*	
H23C	0.9488	0.8753	0.7424	0.162*	
C24	0.7097 (3)	0.6759 (3)	0.7821 (2)	0.0713 (8)	
C25	0.7012 (3)	0.7047 (3)	0.86146 (19)	0.0928 (11)	
H25A	0.6247	0.7072	0.8773	0.111*	
H25B	0.7417	0.6617	0.8927	0.111*	
O7	0.5385 (3)	1.09004 (19)	0.51597 (19)	0.1357 (12)	
C26	0.5766 (4)	0.9442 (3)	0.4603 (2)	0.1191 (15)	
H26A	0.5182	0.9098	0.4369	0.179*	
H26B	0.6277	0.9008	0.4821	0.179*	
H26C	0.6145	0.9819	0.4248	0.179*	
C27	0.5296 (3)	1.0056 (2)	0.5173 (2)	0.0789 (10)	
C28	0.4691 (3)	0.9580 (3)	0.5765 (2)	0.0971 (12)	
H28A	0.4356	1.0042	0.6077	0.146*	
H28B	0.5195	0.9202	0.6045	0.146*	
H28C	0.4127	0.9184	0.5560	0.146*	
H1A	0.451 (2)	0.6847 (18)	0.5023 (14)	0.051 (8)*	
H1B	0.558 (3)	0.676 (2)	0.5390 (16)	0.074 (10)*	

### Atomic displacement parameters (Å^2^)

**Table:**

	*U*^11^	*U*^22^	*U*^33^	*U*^12^	*U*^13^	*U*^23^
Cl1	0.0766 (5)	0.0706 (5)	0.0693 (5)	−0.0249 (4)	0.0075 (4)	−0.0046 (4)
S2	0.0504 (4)	0.0544 (4)	0.0417 (4)	−0.0062 (3)	0.0027 (3)	−0.0028 (3)
O1	0.1156 (17)	0.0536 (12)	0.0607 (13)	−0.0068 (11)	0.0315 (13)	0.0019 (10)
O2	0.1170 (17)	0.0568 (12)	0.0599 (13)	0.0153 (12)	0.0358 (13)	0.0003 (11)
O3	0.0508 (10)	0.0817 (14)	0.0527 (11)	−0.0132 (10)	0.0003 (10)	−0.0086 (11)
O4	0.0678 (11)	0.0693 (11)	0.0459 (10)	−0.0107 (10)	0.0147 (10)	−0.0115 (10)
O5	0.0747 (12)	0.0535 (11)	0.0677 (12)	−0.0128 (10)	0.0109 (11)	−0.0007 (10)
O6	0.0981 (19)	0.0734 (16)	0.143 (3)	−0.0087 (14)	0.0256 (17)	−0.0057 (16)
N1	0.0442 (13)	0.0422 (12)	0.0399 (13)	0.0020 (11)	0.0032 (11)	0.0029 (11)
S1	0.0663 (8)	0.1282 (14)	0.0576 (7)	0.0092 (9)	−0.0056 (6)	0.0178 (8)
C1	0.079 (4)	0.076 (5)	0.052 (3)	−0.006 (3)	0.004 (3)	0.015 (2)
C2	0.060 (3)	0.067 (3)	0.060 (3)	0.022 (3)	0.019 (2)	0.018 (3)
C3	0.056 (4)	0.068 (4)	0.049 (3)	−0.010 (4)	0.003 (3)	0.023 (2)
C4	0.0581 (18)	0.0524 (16)	0.0424 (17)	0.0046 (13)	0.0077 (14)	−0.0041 (13)
S1'	0.065 (4)	0.096 (7)	0.086 (5)	−0.005 (4)	0.023 (3)	0.018 (4)
C1'	0.056 (11)	0.063 (11)	0.054 (10)	−0.006 (8)	−0.002 (8)	0.010 (7)
C2'	0.063 (9)	0.050 (10)	0.036 (8)	−0.025 (7)	−0.002 (7)	0.001 (6)
C3'	0.071 (11)	0.094 (11)	0.065 (11)	0.019 (8)	0.017 (8)	0.028 (8)
C4'	0.0581 (18)	0.0524 (16)	0.0424 (17)	0.0046 (13)	0.0077 (14)	−0.0041 (13)
C5	0.0706 (19)	0.0489 (16)	0.0512 (17)	0.0003 (14)	0.0159 (15)	−0.0015 (13)
C6	0.0604 (16)	0.0541 (15)	0.0456 (15)	−0.0022 (13)	0.0114 (14)	0.0070 (14)
C7	0.0494 (15)	0.0435 (14)	0.0402 (14)	−0.0003 (11)	0.0014 (13)	−0.0018 (13)
C8	0.0573 (15)	0.0470 (16)	0.0443 (15)	0.0071 (13)	0.0043 (14)	0.0038 (14)
C9	0.114 (3)	0.090 (2)	0.063 (2)	0.024 (2)	0.040 (2)	0.0068 (19)
C10	0.0486 (15)	0.0442 (14)	0.0363 (14)	0.0024 (12)	0.0067 (13)	0.0012 (11)
C11	0.0599 (18)	0.0531 (16)	0.071 (2)	0.0039 (14)	−0.0038 (16)	0.0038 (15)
C12	0.066 (2)	0.077 (2)	0.086 (2)	0.0147 (17)	−0.0086 (17)	0.0120 (19)
C13	0.095 (3)	0.069 (2)	0.085 (2)	0.029 (2)	0.008 (2)	0.0193 (18)
C14	0.088 (2)	0.0448 (16)	0.072 (2)	0.0057 (17)	0.0225 (19)	0.0111 (15)
C15	0.0631 (17)	0.0475 (15)	0.0412 (15)	−0.0047 (13)	0.0167 (14)	−0.0008 (13)
C16	0.0644 (17)	0.0670 (18)	0.0516 (16)	0.0198 (15)	0.0014 (14)	−0.0017 (16)
C17	0.0508 (14)	0.0588 (16)	0.0410 (14)	0.0080 (13)	0.0049 (13)	0.0004 (12)
C18	0.074 (2)	0.082 (2)	0.0488 (18)	0.0280 (17)	0.0007 (15)	−0.0035 (15)
C19	0.081 (2)	0.094 (2)	0.067 (2)	0.018 (2)	0.0122 (19)	−0.019 (2)
C20	0.080 (2)	0.110 (3)	0.0417 (16)	0.009 (2)	0.0022 (16)	−0.0083 (18)
C21	0.0567 (17)	0.095 (2)	0.0466 (17)	0.0077 (18)	0.0010 (15)	−0.0069 (17)
C22	0.079 (2)	0.181 (4)	0.072 (2)	0.048 (3)	−0.018 (2)	0.004 (3)
C23	0.085 (2)	0.124 (3)	0.115 (3)	−0.023 (3)	0.015 (3)	−0.036 (3)
C24	0.0628 (19)	0.074 (2)	0.077 (2)	0.0079 (18)	0.0106 (19)	0.0023 (19)
C25	0.093 (3)	0.119 (3)	0.067 (2)	0.017 (2)	0.023 (2)	0.021 (2)
O7	0.187 (3)	0.0668 (16)	0.153 (3)	−0.0252 (18)	−0.005 (3)	0.0062 (18)
C26	0.156 (4)	0.107 (3)	0.095 (3)	0.026 (3)	0.020 (3)	0.021 (3)
C27	0.088 (2)	0.065 (2)	0.084 (3)	−0.0090 (19)	−0.024 (2)	0.005 (2)
C28	0.115 (3)	0.087 (2)	0.090 (3)	−0.009 (2)	−0.008 (2)	−0.007 (2)

### Geometric parameters (Å, °)

**Table:**

Cl1—C15	1.741 (3)	C11—H11	0.9300
S2—O5	1.4418 (18)	C12—C13	1.370 (4)
S2—O3	1.4510 (18)	C12—H12	0.9300
S2—O4	1.4577 (18)	C13—C14	1.369 (5)
S2—C16	1.767 (3)	C13—H13	0.9300
O1—C8	1.188 (3)	C14—C15	1.385 (4)
O2—C8	1.319 (3)	C14—H14	0.9300
O2—C9	1.456 (4)	C16—C17	1.535 (4)
O6—C24	1.210 (4)	C16—H16A	0.9700
N1—C6	1.494 (3)	C16—H16B	0.9700
N1—C7	1.498 (3)	C17—C24	1.519 (4)
N1—H1A	0.88 (3)	C17—C21	1.543 (4)
N1—H1B	0.93 (3)	C17—C18	1.552 (4)
S1—C1	1.706 (5)	C18—C19	1.550 (4)
S1—C4	1.712 (3)	C18—H18A	0.9700
C1—C2	1.333 (6)	C18—H18B	0.9700
C1—H1	0.9300	C19—C20	1.537 (4)
C2—C3	1.472 (7)	C19—H19A	0.9700
C2—H2	0.9300	C19—H19B	0.9700
C3—C4	1.335 (7)	C20—C25	1.501 (5)
C3—H3	0.9300	C20—C21	1.546 (4)
C4—C5	1.504 (4)	C20—H20	0.9800
S1'—C1'	1.709 (10)	C21—C22	1.533 (4)
C1'—C2'	1.338 (9)	C21—C23	1.537 (5)
C1'—H1'	0.9300	C22—H22A	0.9600
C2'—C3'	1.499 (9)	C22—H22B	0.9600
C2'—H2'	0.9300	C22—H22C	0.9600
C3'—H3'	0.9300	C23—H23A	0.9600
C5—C6	1.517 (4)	C23—H23B	0.9600
C5—H5A	0.9700	C23—H23C	0.9600
C5—H5B	0.9700	C24—C25	1.515 (5)
C6—H6A	0.9700	C25—H25A	0.9700
C6—H6B	0.9700	C25—H25B	0.9700
C7—C8	1.514 (3)	O7—C27	1.205 (4)
C7—C10	1.521 (3)	C26—C27	1.474 (5)
C7—H7	0.9800	C26—H26A	0.9600
C9—H9A	0.9600	C26—H26B	0.9600
C9—H9B	0.9600	C26—H26C	0.9600
C9—H9C	0.9600	C27—C28	1.474 (5)
C10—C11	1.385 (3)	C28—H28A	0.9600
C10—C15	1.395 (3)	C28—H28B	0.9600
C11—C12	1.385 (4)	C28—H28C	0.9600
			
O5—S2—O3	113.33 (12)	C14—C15—C10	121.1 (3)
O5—S2—O4	111.76 (11)	C14—C15—Cl1	118.4 (2)
O3—S2—O4	112.61 (11)	C10—C15—Cl1	120.5 (2)
O5—S2—C16	107.82 (14)	C17—C16—S2	118.91 (18)
O3—S2—C16	106.12 (13)	C17—C16—H16A	107.6
O4—S2—C16	104.52 (12)	S2—C16—H16A	107.6
C8—O2—C9	117.2 (2)	C17—C16—H16B	107.6
C6—N1—C7	114.31 (19)	S2—C16—H16B	107.6
C6—N1—H1A	107.9 (16)	H16A—C16—H16B	107.0
C7—N1—H1A	108.2 (16)	C24—C17—C16	112.7 (2)
C6—N1—H1B	111.0 (18)	C24—C17—C21	100.4 (2)
C7—N1—H1B	107.8 (17)	C16—C17—C21	116.5 (2)
H1A—N1—H1B	107 (2)	C24—C17—C18	102.2 (2)
C1—S1—C4	92.6 (2)	C16—C17—C18	120.1 (2)
C2—C1—S1	111.6 (4)	C21—C17—C18	102.2 (2)
C2—C1—H1	124.2	C19—C18—C17	104.2 (2)
S1—C1—H1	124.2	C19—C18—H18A	110.9
C1—C2—C3	112.2 (4)	C17—C18—H18A	110.9
C1—C2—H2	123.9	C19—C18—H18B	110.9
C3—C2—H2	123.9	C17—C18—H18B	110.9
C4—C3—C2	111.9 (5)	H18A—C18—H18B	108.9
C4—C3—H3	124.1	C20—C19—C18	102.5 (2)
C2—C3—H3	124.1	C20—C19—H19A	111.3
C3—C4—C5	126.0 (4)	C18—C19—H19A	111.3
C3—C4—S1	111.5 (4)	C20—C19—H19B	111.3
C5—C4—S1	122.5 (2)	C18—C19—H19B	111.3
C2'—C1'—S1'	111.5 (9)	H19A—C19—H19B	109.2
C2'—C1'—H1'	124.2	C25—C20—C19	106.6 (3)
S1'—C1'—H1'	124.2	C25—C20—C21	102.8 (3)
C1'—C2'—C3'	110.9 (8)	C19—C20—C21	102.6 (3)
C1'—C2'—H2'	124.5	C25—C20—H20	114.5
C3'—C2'—H2'	124.5	C19—C20—H20	114.5
C2'—C3'—H3'	125.2	C21—C20—H20	114.5
C4—C5—C6	111.7 (2)	C22—C21—C23	108.6 (3)
C4—C5—H5A	109.3	C22—C21—C17	113.0 (3)
C6—C5—H5A	109.3	C23—C21—C17	112.7 (3)
C4—C5—H5B	109.3	C22—C21—C20	113.9 (3)
C6—C5—H5B	109.3	C23—C21—C20	113.9 (3)
H5A—C5—H5B	107.9	C17—C21—C20	94.3 (2)
N1—C6—C5	111.0 (2)	C21—C22—H22A	109.5
N1—C6—H6A	109.4	C21—C22—H22B	109.5
C5—C6—H6A	109.4	H22A—C22—H22B	109.5
N1—C6—H6B	109.4	C21—C22—H22C	109.5
C5—C6—H6B	109.4	H22A—C22—H22C	109.5
H6A—C6—H6B	108.0	H22B—C22—H22C	109.5
N1—C7—C8	107.46 (19)	C21—C23—H23A	109.5
N1—C7—C10	112.3 (2)	C21—C23—H23B	109.5
C8—C7—C10	113.4 (2)	H23A—C23—H23B	109.5
N1—C7—H7	107.8	C21—C23—H23C	109.5
C8—C7—H7	107.8	H23A—C23—H23C	109.5
C10—C7—H7	107.8	H23B—C23—H23C	109.5
O1—C8—O2	124.4 (3)	O6—C24—C25	127.1 (4)
O1—C8—C7	123.9 (2)	O6—C24—C17	126.5 (3)
O2—C8—C7	111.6 (2)	C25—C24—C17	106.4 (3)
O2—C9—H9A	109.5	C20—C25—C24	102.3 (3)
O2—C9—H9B	109.5	C20—C25—H25A	111.3
H9A—C9—H9B	109.5	C24—C25—H25A	111.3
O2—C9—H9C	109.5	C20—C25—H25B	111.3
H9A—C9—H9C	109.5	C24—C25—H25B	111.3
H9B—C9—H9C	109.5	H25A—C25—H25B	109.2
C11—C10—C15	117.9 (2)	C27—C26—H26A	109.5
C11—C10—C7	119.8 (2)	C27—C26—H26B	109.5
C15—C10—C7	122.3 (2)	H26A—C26—H26B	109.5
C10—C11—C12	120.9 (3)	C27—C26—H26C	109.5
C10—C11—H11	119.5	H26A—C26—H26C	109.5
C12—C11—H11	119.5	H26B—C26—H26C	109.5
C13—C12—C11	119.8 (3)	O7—C27—C26	122.7 (4)
C13—C12—H12	120.1	O7—C27—C28	121.0 (4)
C11—C12—H12	120.1	C26—C27—C28	116.2 (3)
C14—C13—C12	120.8 (3)	C27—C28—H28A	109.5
C14—C13—H13	119.6	C27—C28—H28B	109.5
C12—C13—H13	119.6	H28A—C28—H28B	109.5
C13—C14—C15	119.4 (3)	C27—C28—H28C	109.5
C13—C14—H14	120.3	H28A—C28—H28C	109.5
C15—C14—H14	120.3	H28B—C28—H28C	109.5
			
C4—S1—C1—C2	−3.8 (6)	O3—S2—C16—C17	−57.0 (2)
S1—C1—C2—C3	2.3 (10)	O4—S2—C16—C17	−176.2 (2)
C1—C2—C3—C4	1.0 (13)	S2—C16—C17—C24	116.6 (3)
C2—C3—C4—C5	177.8 (6)	S2—C16—C17—C21	−128.1 (2)
C2—C3—C4—S1	−3.8 (12)	S2—C16—C17—C18	−3.8 (4)
C1—S1—C4—C3	4.4 (8)	C24—C17—C18—C19	71.6 (3)
C1—S1—C4—C5	−177.1 (3)	C16—C17—C18—C19	−162.9 (3)
S1'—C1'—C2'—C3'	−11 (3)	C21—C17—C18—C19	−32.0 (3)
C3—C4—C5—C6	−101.8 (8)	C17—C18—C19—C20	−3.0 (3)
S1—C4—C5—C6	80.0 (3)	C18—C19—C20—C25	−70.6 (3)
C7—N1—C6—C5	−172.8 (2)	C18—C19—C20—C21	37.0 (3)
C4—C5—C6—N1	−176.6 (2)	C24—C17—C21—C22	66.0 (3)
C6—N1—C7—C8	−178.2 (2)	C16—C17—C21—C22	−55.9 (4)
C6—N1—C7—C10	−52.8 (3)	C18—C17—C21—C22	171.1 (3)
C9—O2—C8—O1	3.1 (4)	C24—C17—C21—C23	−170.4 (2)
C9—O2—C8—C7	−179.7 (2)	C16—C17—C21—C23	67.7 (3)
N1—C7—C8—O1	−6.6 (4)	C18—C17—C21—C23	−65.3 (3)
C10—C7—C8—O1	−131.3 (3)	C24—C17—C21—C20	−52.2 (3)
N1—C7—C8—O2	176.3 (2)	C16—C17—C21—C20	−174.2 (2)
C10—C7—C8—O2	51.6 (3)	C18—C17—C21—C20	52.8 (3)
N1—C7—C10—C11	−67.7 (3)	C25—C20—C21—C22	−62.5 (4)
C8—C7—C10—C11	54.3 (3)	C19—C20—C21—C22	−173.0 (3)
N1—C7—C10—C15	113.0 (3)	C25—C20—C21—C23	172.1 (3)
C8—C7—C10—C15	−124.9 (3)	C19—C20—C21—C23	61.6 (4)
C15—C10—C11—C12	−0.2 (4)	C25—C20—C21—C17	55.0 (3)
C7—C10—C11—C12	−179.5 (3)	C19—C20—C21—C17	−55.5 (3)
C10—C11—C12—C13	0.4 (5)	C16—C17—C24—O6	−20.7 (4)
C11—C12—C13—C14	0.0 (5)	C21—C17—C24—O6	−145.4 (3)
C12—C13—C14—C15	−0.5 (5)	C18—C17—C24—O6	109.5 (3)
C13—C14—C15—C10	0.7 (4)	C16—C17—C24—C25	157.9 (3)
C13—C14—C15—Cl1	−178.6 (2)	C21—C17—C24—C25	33.2 (3)
C11—C10—C15—C14	−0.4 (4)	C18—C17—C24—C25	−71.8 (3)
C7—C10—C15—C14	178.9 (2)	C19—C20—C25—C24	71.9 (3)
C11—C10—C15—Cl1	179.0 (2)	C21—C20—C25—C24	−35.6 (3)
C7—C10—C15—Cl1	−1.8 (3)	O6—C24—C25—C20	179.9 (3)
O5—S2—C16—C17	64.8 (2)	C17—C24—C25—C20	1.3 (3)

### Hydrogen-bond geometry (Å, °)

**Table:**

*D*—H···*A*	*D*—H	H···*A*	*D*···*A*	*D*—H···*A*
N1—H1B···O3	0.93 (3)	1.82 (3)	2.729 (3)	164 (3)
N1—H1A···O5^i^	0.88 (3)	2.63 (2)	3.180 (3)	121.1 (19)
N1—H1A···O4^i^	0.88 (3)	1.99 (3)	2.856 (3)	169 (2)

Symmetry codes: (i) *x*−1/2, −*y*+3/2, −*z*+1.
